# Three-Dimensional Bioprinting in Soft Tissue Engineering for Plastic and Reconstructive Surgery

**DOI:** 10.3390/bioengineering10101232

**Published:** 2023-10-21

**Authors:** Astrid Bülow, Benedikt Schäfer, Justus P. Beier

**Affiliations:** Department of Plastic Surgery, Hand Surgery, Burn Center, University Hospital RWTH Aachen, 52074 Aachen, Germany; bschaefer@ukaachen.de (B.S.); jbeier@ukaachen.de (J.P.B.)

**Keywords:** tissue engineering, 3D bioprinting, skeletal muscle tissue engineering, adipose tissue

## Abstract

Skeletal muscle tissue engineering (TE) and adipose tissue engineering have undergone significant progress in recent years. This review focuses on the key findings in these areas, particularly highlighting the integration of 3D bioprinting techniques to overcome challenges and enhance tissue regeneration. In skeletal muscle TE, 3D bioprinting enables the precise replication of muscle architecture. This addresses the need for the parallel alignment of cells and proper innervation. Satellite cells (SCs) and mesenchymal stem cells (MSCs) have been utilized, along with co-cultivation strategies for vascularization and innervation. Therefore, various printing methods and materials, including decellularized extracellular matrix (dECM), have been explored. Similarly, in adipose tissue engineering, 3D bioprinting has been employed to overcome the challenge of vascularization; addressing this challenge is vital for graft survival. Decellularized adipose tissue and biomimetic scaffolds have been used as biological inks, along with adipose-derived stem cells (ADSCs), to enhance graft survival. The integration of dECM and alginate bioinks has demonstrated improved adipocyte maturation and differentiation. These findings highlight the potential of 3D bioprinting techniques in skeletal muscle and adipose tissue engineering. By integrating specific cell types, biomaterials, and printing methods, significant progress has been made in tissue regeneration. However, challenges such as fabricating larger constructs, translating findings to human models, and obtaining regulatory approvals for cellular therapies remain to be addressed. Nonetheless, these advancements underscore the transformative impact of 3D bioprinting in tissue engineering research and its potential for future clinical applications.

## 1. Introduction

Plastic and reconstructive surgery deals with many different conditions affecting different types of tissue. The goal of reconstructive surgery is to restore physical integrity in terms of aesthetics and functional aspects. This includes, for example, reconstructive measures after nerve injuries or the loss of skeletal musculature.

Currently, there are several surgical options available to treat significant tissue loss. The type of treatment varies depending on the type of tissue involved and whether functional loss needs to be treated. The medical gold standard for the treatment of skeletal muscle or significant soft tissue defects is autologous muscle or tissue transplantation. In the case of a soft tissue defect without functional limitations, e.g., after an open fracture with exposed bone, transplantation of a fasciocutaneous flap may be sufficient. If a functional muscular defect exists, transplantation of skeletal muscle is performed with intact blood and nerve supply. The in-growth of nerves into the transplanted tissue is a major challenge. Complete functional regeneration of the transplanted muscle at the recipient site is rarely achieved [1]. In the absence of functional regeneration after transplantation, secondary procedures can be performed to restore function, such as tendon transfers. This is where the tendons of still-functioning muscles are transferred to the tendons of denervated or injured muscles [2]. Transplantation of autologous adipose tissue is also commonly performed for defect reconstruction. However, this often results in the resorption of a large proportion of the transplanted tissue [3]. Although there are surgical approaches by which to treat the various tissue defects, each with a different prognosis, all techniques have one major disadvantage in common. Any transplantation of autologous tissue inevitably results in a harvesting defect and donor site morbidity. The transplantation of skeletal muscle leads to a loss of its specific muscle function at the donor site. In addition, due to the poor vascular status of the patient and the localization or special requirements of a defect, suitable autologous tissue for transplantation or a suitable recipient site may not be available. For this reason, soft tissue engineering is of great importance for plastic and reconstructive surgery.

The aim of tissue engineering (TE) is to create an equivalent tissue substitute to avoid the comorbidities of autologous transplantations and to achieve the best possible results in the case of functional reconstruction. For this purpose, there are different approaches, which are based on three basic building blocks [4]. First, various progenitor cells are used to differentiate and form new tissue. In addition, growth factors and biophysical stimuli play an important role in the control of cell differentiation and development [5,6]. Finally, carrier structures or scaffolds are required, which imitate the extracellular matrix of the intended tissue. They should provide the cells with an optimal environment for proliferation and differentiation. Above all, any engineered soft tissue construct must be sufficiently vascularized to engraft successfully and permanently at the recipient site. Different strategies, such as in vitro prevascularization, on-site proangiogenic factor release, or donor-site-based in vivo prevascularization (e.g., AV-loop, AV-bundle), have been proposed [7,8].

In this review, we focus on conventional scaffold-based technology and its evolution to 3D bioprinting approaches for TE and regeneration of the two soft tissues that are most important in plastic and reconstructive surgery: skeletal muscle tissue and adipose tissue.

Scaffolds are used as acellular implants to promote and guide the endogenous tissue regeneration process. Alternatively, scaffolds can be used as cell-laden constructs, meaning they contain cells that are intended to differentiate and form the tissue of interest.

There are several key requirements that scaffolds must meet in order to be effective. First, they must be biocompatible, meaning they do not elicit an adverse immune response or cause other harmful effects when introduced into the body. They must also support cellular attachment, meaning that cells are able to adhere to the scaffold and grow on it. Scaffolds are not meant to be permanent implants. They are designed to be degraded over time and replaced by endogenous tissue [4]. In addition, the mechanical properties of the scaffold must correspond as closely as possible to those of the desired tissue. 

In addition to these structural properties, scaffolds must be permeable and conductive to support the in-growth of blood vessels and the transmission of signals.

Scaffolds for the TE of soft tissues have been made using a wide range of biomaterials, which can generally be divided into two main categories: natural biomaterials and synthetic biomaterials. Natural biomaterials derive from biological sources and may mimic the native extracellular matrix more closely. Some of the most frequently used natural biomaterials are collagen, gelatin, and alginate. However, natural biomaterials have some limitations, including batch-to-batch variability. Furthermore, these materials often exhibit poor mechanical properties and fast degradation in vivo. Synthetic biomaterials, such as poly(ε-caprolactone) (PCL), polylactic acid (PLA), and polyurethane (PU), can be designed more precisely and have good mechanical and nanoscale properties. However, they may not be as biocompatible or support good cell adhesion, and they can lack the bioactivity of some natural biomaterials like collagen or gelatin. Additionally, many degradation products of these polymers consist of acidic compounds that can cause undesirable immune reactions [9]. To overcome the limitations of both types of materials, it is common to use combinations of different natural and synthetic materials in scaffold design. For example, a scaffold made from synthetic materials can be combined with a cell-loaded hydrogel to take advantage of the benefits of both types of materials [10]. Another emerging approach is the use of silk fibroin or nanocellulose as a biomaterial. These materials combine the advantageous physiochemical properties of other natural biomaterials, such as biodegradation and biocompatibility, with the mechanical advantages of synthetic materials. Furthermore, both materials are printable. In addition, silk fibroin has been shown to promote cellular behavior and host-implant integration [9,11,12,13]. 

**Figure 1:**
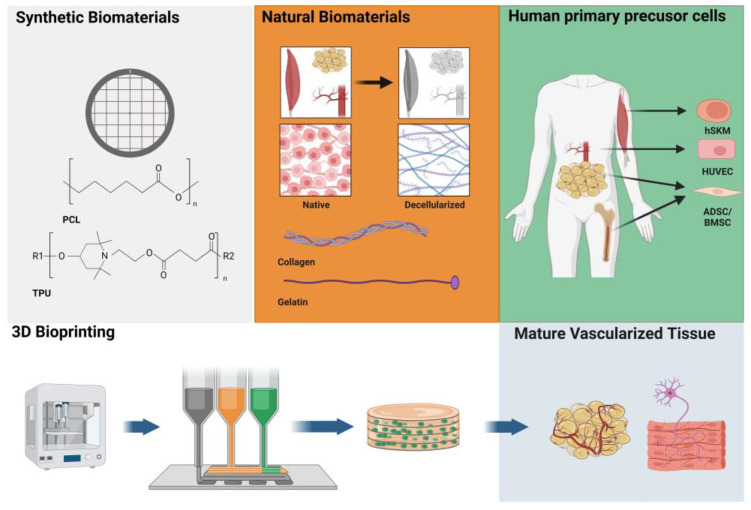
Schematic representation of the principles and advantages of bioprinting. Different components, such as cells or structuring materials, can be used as ink and printed simultaneously. The aim is to grow mature tissue for implantation in humans. PCL—polycaprolactone; TPU—thermoplastic polyurethane; hSKM—human skeletal muscle cells/satellite cells; HUVECs—human endothelial cells; AD/BMSCs—adipose/bone-marrow-derived mesenchymal stem cells. Created with BioRender.com.

In this review, we discuss the latest developments in soft tissue engineering using 3D bioprinting as the manufacturing method. In recent years, the importance of 3D bioprinting in tissue engineering has increased significantly. The major advantage of 3D bioprinting over other commonly used manufacturing methods for creating 3D scaffolds, including freeze-drying and electrospinning, is that it allows precise control of the internal architecture and topology [14,15]. It also enables a high degree of reproducibility and accuracy. Another advantage of 3D bioprinting is that the different components needed to create a scaffold for complex tissue reconstructions can be printed simultaneously and in a controlled manner. In the case of a cellular scaffold, it is possible to print a structuring material and the tissue-specific progenitor cells, mesenchymal stem cells, and biological factors, e.g., encapsulated in a hydrogel, simultaneously [5,6]. Thereby, a controlled distribution of cells in the scaffold is possible, circumventing the difficulties of colonization, especially of larger constructs (Figure 1) [16]. Although the technique’s beneficial utilization has been proven in the production of several tissues, a major challenge remains in the production of soft, elastic, and dynamic tissues [17]. For bone and cartilage tissue engineering, the individual clinical applications of 3D-printed constructs have already been described [18,19]. One challenge of 3D bioprinting is that shear forces or thermal effects can damage cells during the printing process. Research has been ongoing to improve the technology for its use in tissue engineering. Commonly used forms of 3D printing are inkjet and extrusion-based bioprinting. In inkjet printing, the printing is completed in the form of drops, while in extrusion-based printing, the printing is executed in a continuous stream. In addition, there are other techniques, e.g., laser-based or photo-polymerization-based. Each technique has its own advantages and disadvantages [20]. The technical details will not be discussed in this review and have been summarized elsewhere [14,15]. 

## 2. Skeletal Muscle Tissue Engineering

**Figure 2:**
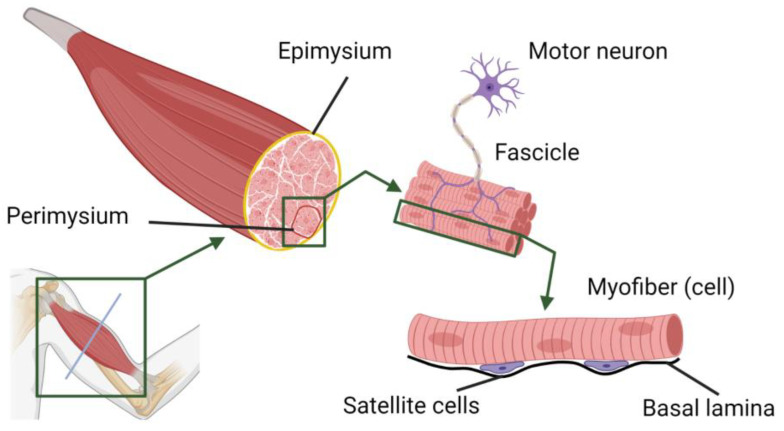
Schematic representation of the anatomy of skeletal muscle. The muscle is shown as consisting of multiple fascicles surrounded by the epimysium. The fascicles, in turn, consist of several myocytes or myofibers enclosed by the perimysium. Myofibers are multinucleated cells. Satellite cells (muscle progenitor cells) lay between the cell membrane of the myocytes (sarcolemma) and the basal lamina. Created with BioRender.com.

The unique challenge of skeletal muscle TE results from the anatomy of the musculature. One muscle consists of multiple myofibers, or myocytes, which are post-miotic, multinucleated cells. Groups of those cells are embedded in connective tissue called the perimysium. A layer of connective tissue called the epimysium in turn surrounds several groups of these bundles, forming the muscle. Each myofiber is innervated by a single motor axon, whereas the motor axon can innervate several myofibers (Figure 2) [21]. In order to obtain a functioning muscle, there must be a parallel, uniaxial alignment of the cells. However, innervation of the muscle and vascularization must be ensured. Scaffolds for skeletal muscle engineering should mimic the native extracellular matrix and include biophysical or biochemical cues to support the formation of new muscle tissue. Therefore, they need to be elastic and withstand heavy uniaxial loads at the same time. The material should stretch up to 60% before mechanical failure [22]. Gotti et al. [23] reported the core features of native skeletal muscle: the failure stress was reported in the range of 70–800 kPa, with a failure strain of 30–60% and an elastic modulus of 30–8000 kPa. Due to the high accuracy of 3D bioprinting, this method seems to be the most suitable to create the complex architecture necessary for successful skeletal muscle TE. Conventional techniques, such as electrospinning, have also been used to produce skeletal muscle constructs with aligned cells. Nevertheless, those scaffolds are usually limited to the fabrication of realistic, large, 3D muscle grafts [24]. With respect to larger constructs, perhaps the greatest disadvantage of techniques, such as electrospinning or directed freeze-drying, is the need for secondary colonization. Achieving homogeneous colonization is a challenge in this regard. In addition, while co-cultivation with different cell types, like endothelial and skeletal muscle progenitor cells, is possible, there is no precise spatial distribution of the colonized cells [25,26]. Using 3D bioprinting, it is possible to create large tissue constructs with complex geometries by using cell-laden hydrogels in a layer-by-layer fashion in combination with biomaterials that serve as structuring analogs of the extracellular matrix [27,28,29]. However, the search for the most suitable biomaterial that meets the requirements mentioned above is still ongoing. 

### 2.1. Cellular Aspects of Skeletal Muscle TE

In vitro TE is mainly based on the cultivation and differentiation of satellite cells (hSKMs) on the cellular side. These are tissue-specific progenitor cells in skeletal muscle. SCs are localized in a niche between the basement membrane and the cell membrane of the myofibrils and the sarcolemma and are in the quiescent phase of the cell cycle [30]. In response to stress or in the context of trauma, they are activated, leave their niche, and enter the cell cycle. In this process, hSKMs differentiate into myoblasts, which eventually fuse into multinucleated myocytes and can contribute to muscle growth or muscle regeneration after injury to a limited extent [31,32]. Another cell population used in skeletal muscle TE is mesenchymal stem cells (MSCs). MSCs are multipotent progenitor cells that are found in the stroma of various tissue types (e.g., umbilical cord, adipose tissue) and can differentiate into multiple cell lineages of the mesodermal lineage, including adipocytes, osteoblasts, chondrocytes, or myocytes [33,34,35]. The most commonly used tissues for the isolation of MSCs are bone marrow (BMSCs) and adipose tissue (ADSCs). However, the use of BMSCs has become less common in recent years due to the limitations in obtaining BMSCs, mainly reflected in the risk of morbidity associated with bone marrow aspiration and the extremely low yield of isolated MSCs (0.001–0.01% of aspirated bone marrow cells) [36]. In contrast, obtaining MSCs from adipose tissue is convenient and efficient. Adipogenic MSCs (ADSCs) can be isolated in high numbers from intraoperatively obtained adipose tissue or lipoaspirate after liposuction. In addition to the advantage of easier isolation, it has been shown that adipogenic MSCs have a higher myogenic potential than BMSCs [37,38]. Furthermore, co-cultivation with endothelial cells [39,40] or neuronal cells has been performed to improve the vascularization or innervation of the muscle, respectively [41,42].

For proof-of-principle, most studies engage another cell type, which is a murine immortal myoblast cell line called C2C12 [43]. The cell line is easier to culture and abundant, unlike primary cells. Ultimately, primary cells are necessary not only for translation but also for pivotal studies in which large animal models, such as pigs, are used. Unlike rat models, where immunodeficient species like the nude rat can be used, autologous cells are mandatory when using large animal models to avoid an immune response [44].

### 2.2. Three-Dimensional (Bio) Printing and Skeletal Muscle TE

Three-dimensional printing is used for skeletal muscle TE in several ways. First, it is used for the sole production of scaffolds, where 3D printing is superior to conventional methods in terms of replicating the architecture of skeletal muscle as accurately as possible. Additionally, the various components (biomaterials, cell types, and growth factors) already mentioned can be combined and the printing process unified.

Gokyer et al. [45] evaluated a novel 3D-printed scaffold as an acellular and cell-laden scaffold in direct comparison in an in vivo model. To produce a scaffold with properties as similar as possible to skeletal muscle ECM in terms of elasticity and stiffness, they developed a novel thermoplastic polyurethaneurea elastomer. Thermoplastic polyurethane and polyurethaneurea copolymers (TPU) consist of alternating soft and hard segments covalently bonded to each other along a linear macromolecular backbone. In contrast to other often-used synthetic biomaterials like PCL, TPU is a more elastic and deformable biomaterial that meets the requirements of skeletal muscle. The analysis showed 940% elongation for the novel TPU scaffold, in contrast to 28% for PCL. The scaffold was printed with a parallel-aligned internal architecture. After in vitro experiments, where the scaffold was successfully seeded with C2C12 cells and human-adipose-tissue-derived MSCs (hADSCs) and showed differentiation of the C2C12 cells, the scaffolds were inserted in an acellular manner or cell-laden with hADSCs in a rat volumetric muscle loss (VML) model. In the cell-laden scaffold, they were able to show regeneration of muscle tissue in contrast to the acellular scaffold or the control group. Moreover, the regenerated muscle proved to be functional with an increase in force generation as compared to the preoperative state (112%), which is a very promising result.

A different method used to find the ideal material to mimic the native extracellular matrix (ECM) is based on the native extracellular matrix itself in the form of decellularized ECM (dECM). It retains important components of the native tissue, e.g., growth factors, and thereby improves tissue regeneration or differentiation of encapsulated cells [46]. After decellularization, dECM can be further processed into a hydrogel. Kiratitanaporn et al. [47] took advantage of the beneficial characteristics of dECM. They developed the synthesis of poly(glycerol sebacate) acrylate (PGSA) as a biocompatible, degradable, and printable material for a skeletal muscle construct. Printing was performed with a DLP/light-based bioprinter. Through modification of the light intensity during the printing process, the mechanical properties of the PGSA changed and could be adjusted. The stiffness of the scaffold was adjusted to the specific tension of skeletal muscle (107–225 kPa). The PGSA scaffold was then coated with dECM derived from porcine skeletal muscle (skmdECM). The coating with skmdECM increased cell infiltration, proliferation, and maturation in an in vitro model using C2C12 cells as well as in an in vivo rat VML model. In vivo, the scaffold showed a lower rejection reaction when coated with skmdECM. The non-coated scaffold was encapsulated in a fibrous ring 28 days after implantation that was not present around the coated scaffold. Despite these promising results and good cell infiltration of the construct, there was little new skeletal muscle tissue formation. 

In addition to the coating of scaffolds, dECM hydrogel can be used as a bioink itself. The printing of the cells in a hydrogel, e.g., from dECM, simultaneously with the scaffold allows accurate distribution of the cells and improved colonization of the support scaffolds. Choi et al. [48] used a hydrogel as a bioink derived from porcine skeletal muscle tissue. C2C12 cells were encapsulated in the gel and printed in differently shaped constructs that were framed in PCL. The research team was able to control the shapes, pores, and architectures with high accuracy and achieve alignment of the cells along the longitudinal axis. They also demonstrated successful myogenic differentiation of the cells, which was detected using immunofluorescence and qPCR. In that study, the construct printed with skmdECM was compared to a control construct consisting of collagen only and showed significantly better results in regard to differentiation, proliferation, and mechanical properties. The construct with skmdECM had an elastic modulus of 12 +/− 3 kPa, which is similar to native muscle (ca. 12 kPa), after 14 days of cultivation, in contrast to the collagen construct with ca. 4 kPa. The fusion index as a marker for differentiation of the myoblast was ca. 55% (skmdECM) vs. 30% (collagen). Other studies support the superiority of skmdECM compared to collagen or other natural components [49,50]. Kim et al. [49] showed that numerous growth factors and cytokines are preserved in the skmdECM and are responsible for the positive effect of the skmdECM. 

These studies show promising results for the use of 3D bioprinting in skeletal muscle TE. However, some important aspects remain to be addressed for clinical translation. Russel et al. [51] have provided a solution to a practical problem. They created a handheld partially automated bioprinter, which is an extrusion-based device capable of continuously extruding biomaterials and includes an integrated light source for crosslinking the extruded bioink for treatment of VML in situ. As a bioink, they developed and characterized a photocrosslinkable gelatin methacryloyl hydrogel. The characterization showed that the mechanical properties of the hydrogel were comparable to those of skeletal muscle. It was also suitable for encapsulating C2C12 cells. The printing process with the developed handheld device did not induce significant cell death. In vitro, the differentiation of the C2C12 cells was successfully induced. For the in vivo evaluation, the gel was used as an acellular scaffold in a VML murine model with a significant defect in the quadriceps muscle. Four weeks after implantation, however, there was no regeneration of muscle tissue. An important advance was made with the development of a handheld device that allows printing directly in situ. However, the hydrogel used in the study, at least as an acellular construct, does not seem to be suitable for the treatment of VML. 

A more pressing problem with respect to 3D-engineered muscles is vascularization. The maximum nutrient and oxygen diffusion distance for cells to survive without vascularity is approximately 200 µm; therefore, constructs may not exceed 1 mm in diameter (or thickness) without additional strategies for nutrient supply to the cells [52,53]. At this scale, the constructs are not usable for clinical applications in humans. A promising approach is the pre-vascularization of scaffolds via co-cultivation with endothelial cells [39,40,54].

Following implantation of such a construct in vivo, the preformed vessels can anastomose with the local vessels. Additionally, neovascularization of the construct is increased via paracrine signaling pathways. Choi et al. [55] used co-cultivation with endothelial cells. They developed a 3D-printed construct consisting of skeletal muscle dECM and endothelial dECM (based on aortic tissue, vdECM) loaded with human muscle and endothelial cells. The construct showed remarkable results when tested in vivo in a rat VML model. The construct successfully mimicked the hierarchical architecture of vascularized muscle and demonstrated improved de novo muscle fiber formation, vascularization, and innervation. It also showed the importance of the spatial arrangement of the cells. The team tested three different constructs in vivo: one consisting of muscle cells, one consisting of muscle and endothelial cells randomly mixed, and the last involving coaxial printing and spatial arrangement of the cells. The coaxial-printed scaffold was superior in regard to the number of blood vessels (350 vs. 220 vs. 140) as well as functional recovery in VML injuries (85% vs. 70% vs. 60%). Choi et al.’s study once again emphasizes the important progress in skeletal muscle TE using 3D bioprinting. Nevertheless, the muscle construct they developed is still no larger than a few centimeters. Even the technique of pre-vascularization, which has been known and used for many years, has not yet led to the development of muscle constructs in a size relevant for human application. 

Another method, which also includes muscle tissue innervation, is the use of a neurotized AV loop or EPI loop model. The EPI loop is based on the saphenous artery and the superficial inferior epigastric veins, as well as the obturator nerve [42]. The combination of these techniques seems to be a promising approach. 

In conclusion, 3D bioprinting has led to many advances in the field of skeletal muscle TE (Table 1). As a fabrication method, it has many advantages that are of great benefit, specifically for the TE of skeletal muscle tissue, due to the high complexity and requirement for the successful TE of functional skeletal muscle tissue as discussed. However, one problem that remains is the fabrication of larger constructs and the associated adequate vascularization. Furthermore, most studies have been performed using the C2C12 cell line, as the isolation and cultivation of primary human myoblasts or satellite cells is a major challenge. As a proof-of-principle or model for myoblasts, the cell line certainly serves its purpose, but for a potential clinical application, these cells are obviously not suitable. Further, cellular constructs have been shown to be superior to acellular constructs in terms of skeletal muscle tissue regeneration, so further research using human cells is urgently needed in this respect. Meanwhile, this is a disadvantage for skeletal muscle TE, as cellular therapies pose a major challenge for clinical translation in terms of potential approval by regulatory bodies such as the FDA (Food and Drug Administration) or EFSA (European Food Safety Authority).

## 3. Adipose Tissue Engineering

Lack of adipose tissue due to trauma or tumor resections is a common challenge in plastic surgery. This can be due to an aesthetic deficit or a functional deficit such as a shifting layer, e.g., in exposed tendons. In addition to flap grafting, autologous fat transfer is one of the most frequently performed operations. Unfortunately, this essentially simple method of transplanting cells in a cell suspension gained by liposuction has the major disadvantage that about 40–80% of the transplanted cells, which mainly consist of mature adipocytes, become necrotic (or apoptotic) and degrade due to insufficient angiogenesis and thereby vascularity [3]. It has already been shown in studies with small numbers of patients that the enrichment and transplantation of adipogenic mesenchymal stem cells or the stromal vascular fraction leads to an improvement in graft survival [58,59,60]. Other approaches used to overcome the lack of vascularization are tissue engineering and the development of scaffolds, which allow vascularization via a controlled structure and pore size or via prevascularized scaffolds [61]. In addition, biomimetic scaffolds are known to promote stem cell growth and differentiation. For these reasons, 3D bioprinting is also used in the TE of adipose tissue, even though the structure of the tissue itself does not play a major role in functionality, unlike, for example, skeletal muscle tissue (Table 2).

Säljö et al. [62] combined the already-used method of purification of lipoaspirate with bioprinting and used the mechanically purified lipoaspirate as the basis for a 3D-printed construct. For use as a biological ink, it was mixed with alginate and nanocellulose. The construct was implanted in mice, and long-term survival was studied. The constructs retained their size and shape over a period of 150 days. However, histological studies showed that the portion of adipose tissue in the engineered tissue decreased from day 0 to day 150 and was replaced by fibrosis. A quantitative evaluation was not provided. It was shown that mechanically processed lipoaspirate can be bioprinted into a customized 3D size and shape. However, the preservation of shape comes at the expense of tissue quality when fibrotic tissue develops. In addition, a control group with a comparison to a non-printed conventional lipotransfer was not included in the study, so it is not possible to make a statement regarding the superiority of 3D bioprinting.

Pati et al. [63] developed a scaffold made of human decellularized adipose tissue (hdECM), which was used as a biological ink. The scaffold was also made of PCL to achieve a stable and porous structure. This led to a problem, as the scaffold was significantly stiffer than native adipose tissue (compressive modulus of the printed constructs: 122.56 ± 20.23 kPa vs. native fat tissue: 19 ± 7 kPa). Human ADSCs were used for the colonization. In vitro testing showed good long-term survival of the cells as well as successful differentiation, with the cells in the printed construct showing significantly increased differentiation compared to the dECM gel. The fold change of expression of PPARγ after 14 days was ca. 10 × in the bioprinted construct in comparison to the dECM gel with ca. 5 ×. In vivo testing was performed, where acellular constructs (PCL alone, PCL + dECM) and cellular constructs (PCL + dECM + hADSC, dECM gel + hADSC not printed) were implanted into a mouse model. The dECM in general showed a proangiogenic effect. However, the dECM gel was completely degraded after 12 weeks. The PCL scaffold showed marked fibrosis. The other two constructs showed good integration into the host tissue. In addition, some cells were PPARγ-positive as a sign of adipogenic differentiation. Pati et al.’s study exemplifies the positive effects of dECM and the use of 3D bioprinting in fat TE.

The use of dECM for adipogenic TE is widespread due to its positive effect on ADSC proliferation and differentiation [64]. Ahn et al. [65] also developed a bioink based on human adipogenic dECM. To achieve densely packed and organized proliferation of cells and thus successful differentiation, the authors developed an in-bath printing technique using a bioink with a cell-friendly environment for cell encapsulation (dECM) and a hybrid bioink with a cell-unfriendly environment for the bath suspension (alginate). The clear superiority of this printing technique over conventional TE methods (direct printing, 2D culture) was demonstrated with respect to adipocyte maturation and differentiation. The environmentally controlled in-bath 3D-bioprinted group had the largest lipid droplet size at 46.1 +/− 8.7 µm (vs. 7.3, 11.3, and 30.4 µm, respectively) and significantly enhanced adiponectin secretion at 294.6 ng/mL compared to 95.5, 134.8, and 194.1 ng/mL, respectively. Excitingly, the authors also performed a functional test of the cultured adipose tissue, simulating obesity. This resulted in similar pathological changes in vitro, which have been shown in studies of obesity in humans (hypertrophy, increased intracellular triglyceride levels, insulin resistance). Thus, the group succeeded in culturing functional adipose tissue, which seems suitable as an in vitro model for obesity research or other adipose-tissue-related diseases. The application of regenerative medicine seems to be rather secondary due to the low dimensions of the tissue. 

Another printing technique that has been proposed for adipogenic TE is indirect printing. Van Damme et al. [66] used the technique, creating a negative mold of the desired scaffold from sacrificial material into which the target material was printed. Biological testing of the scaffold has not yet been performed. Negrini et al. [67] also used indirect printing with sacrificial structures to improve vascularization. They implemented 3D-printed alginate filaments in a gelatin hydrogel together with alginate microbeads as sacrificial structures. The channel formed by the alginate filament is thought to be suitable for vascularization. The scaffolds showed pores with diameters ranging from 200 to 400 µm, as well as 93% porosity because of the sacrificial microbeads. The mechanical properties obtained for the scaffold were comparable in terms of the elastic modulus (3.7 kPa vs. 2.6 kPa native tissue) and the maximum stress, representing the stress at the highest compression strain (1.5 kPa vs. 1.8 kPa), to those measured for subcutaneous native adipose tissue. The in vitro analysis showed successful colonization and differentiation of hMSCs on the scaffold. However, in vitro testing was performed to investigate the general compatibility in terms of cell viability and differentiation without using the created channel, e.g., via co-cultivation with endothelial cells. Additionally, the team demonstrated in an ex vivo study a possible mechanism of implanting the scaffold with the suture of a rat vessel to the preformed channel/vessel in the hydrogel. 

The studies listed above demonstrate the application of 3D bioprinting to adipose TE. Again, the method shows advantages, including high control of the shape of the construct and good mimicking of the ECM of the target tissue, especially in combination with a decellularized extracellular matrix as a hydrogel. However, in contrast to skeletal muscle TE, 3D printing does not stand out as much compared to other conventional techniques, such as cell seeding scaffolds or hydrogels [68]. This may be mainly due to the less complex anatomy and the lower requirements for a scaffold to maintain the functionality of the adipose tissue. However, in adipose tissue TE, a major challenge is the fabrication of larger and thus vascularized constructs. In particular, when co-culturing with endothelial cells to produce a prevascularized scaffold or integrating defined macropores, 3D bioprinting shows advantages in its precise spatial arrangement and reproducibility [69]. The importance of 3D bioprinting in the field of adipose tissue engineering will increase, according to the authors of this review.

**Table 2:** Summary of the reviewed literature related to adipose tissue engineering.

Paper	Cell Type	Bioink	Experiment Type	Key Findings	Limitations
Pati et al. (2015) [63]	hADSC	PCL + hdECM	In vitro	Successful cultivation of the cells and differentiation	Mechanical properties do not match adipose tissue
			In vivo murine VML model	dECM showed proangiogenic effect, printed scaffold superior over non-printed	Small size of construct
Ahn et al. (2022) [65]	hADSC	hdECM + Alginate	In vitro	In-bath hybrid printing technique superior to 3D printing; culturing of functional adipose tissue	Low dimensions of the tissue, no in vivo application
Lee et al. (2021) [64]	-	PCL + mixture of collagen type I and hdECM hydrogels	In vivo murine model	hdECM hydrogel promotes neovascularization and tissue formation	Small size of construct
Van Damme et al. (2020) [66]	-	GelMa + PLA (sacrificial)	In silico	Comparison of indirect vs. direct printing technique -> similar results regarding mechanical properties	No biological testing
Negrini et al. (2019) [67]	hMSC *	Alginate microbeads (sacrificial) MBA crosslinked gelatin hydrogel	In vitro	Microporous gelatin hydrogels, suitable as scaffolds for AT (porosity, mechanical properties, enzymatic degradability, and hMSC proliferation and differentiation)	In vivo application pending
			Ex vivo	Perfusable vascular channel in the scaffold	
Negrini et al. (2020) [70]	hADSC	MBA crosslinked gelatin hydrogel	In vitro	Physical and mechanical properties for use as AT scaffolds Support cell proliferation and differentiation	In vivo application pending
Säljö et al. (2022) [62]	Stroma vascular fraction	Alginate and nanocellulose	In vivo murine model	Printability of mechanically purified lipoaspirate and in vivo long-term survival	Control group missing, formation of fibrotic tissue rather than mature adipose tissue

* cell origin not stated; MBA—methylenebisacrylamide.

## 4. Vascularization

In addition to the clinical need to replace blood vessels, e.g., in the context of bypasses, a particular challenge in tissue engineering is the vascularization of newly engineered tissue, as mentioned previously. The cultivation of different tissue types is already succeeding. For the development of larger constructs, which are thus relevant for therapy, it is important to create a 3D vascular-like network within the engineered tissue. There are already several approaches. These include the use of proangiogenic growth factors in the constructs, prevascularization, or the formation of vascular channels or networks via direct or indirect manufacturing methods [71,72]. In addition, there is an approach to using O_2_-generating materials partially as encapsulated O_2_ sources in other commonly used materials such as PCL or PLGA to ensure cell viability within the construct [73]. Three-dimensional bioprinting has also enabled significant progress in this area (Table 3). 

Referring to the last mentioned technique of O_2_-generating materials, Erdem et al. [74] developed a bioink that consists of GelMa and calcium peroxide (CPO) as an O_2_ source for the nutrient supply of the printed cells. They cultivated fibroblasts and rat cardiomyocytes under normoxic and hypoxic conditions in the construct, with similar cell viability in the hypoxic group when CPO was added to the bioink. 

Kreimendahl et al. [75] investigated the vascularization of a fibrin-hyaluronic acid construct (Fibrin-HA) using an innovative printing technique (FRESH, Freeform Reversible Embedding of Suspended Hydrogels Bioprinting). This technique involves bioinks being extruded into a supportive gelatin bath to prevent hydrogel constructs from collapsing and deforming during the printing process. It enables the printing of highly complex 3D structures with diverse low-viscosity bioinks like fibrin and other natural polymers. The authors printed Fibrin-HA constructs encapsulating HUVECs and human dermal fibroblasts (HDFs) and were able to show 87% viability after printing, as well as the formation of a vascular network in the construct. 

Sousa et al. [76] used 3D printing as an additive manufacturing method to fabricate the sacrificial structures of alginate, which were inserted into a scaffold based on a glycidyl methacrylated xanthan gum (XG-GMA)-based scaffold, ultimately forming microchannels. Three-dimensional printing makes it possible to print different geometries of the sacrificial structures easily and reproducibly and thus achieve a complex internal architecture of the subsequent hydrogel scaffold. In [74], the microchannels were successfully colonized with human endothelial cells in vitro (HUVEC). 

This fabrication technique can imitate a complex vascular network. However, it requires several steps and the combination of different techniques. Shao et al. [77] developed a scaffold fabricated by simultaneously printing sacrificial structures and cell-loaded bioink. For this, they used gelatin as the sacrificial material and GelMa for the scaffold itself. They took advantage of the fact that both materials can be thermally reversibly crosslinked. For permanent stability, GelMa was subsequently photocrosslinked while the gelatin structures dissolved during cultivation at 37 °C, creating a nutrient network. By adjusting the flow rate of the two components, the pore size and density of the network could be influenced. The shape of the construct was also highly variable. Colonization with HUVECs and osteoblasts (mouse cell line MC3T3) was successful. Even in constructs with a size of 3 × 3 × 3 cm^3^, the networks were able to achieve good viability of the cells in the center of the constructs in vitro. Differentiation was not investigated. 

Kolesky et al. [78] employed a similar production technique to produce thick, vascularized tissue. Silicone was used to create the frame for the vascularized tissue. Alternate printing of a vascular network of sacrificial or fugitive ink (Pluronic F-127 and thrombin) and a network of cell-loaded ink (gelatin, fibrinogen, and target cells) followed this. Finally, casting of ECM (gelatin, fibrinogen, thrombin, support cells, and transglutaminase) was performed. By cooling the construct, the fugitive ink liquefied and was evacuated. The network was then endothelialized via perfusion with HUVECs. The authors demonstrated the formation of endothelialized hollow vascular structures in the construct, which ensured adequate nutrient supply to the cells in the construct. A construct with a volume of 10 cm³ containing hMSCs was prepared. Osteogenic differentiation of stem cells was induced via perfusion with an osteogenic differentiation medium, which was also successfully demonstrated in the center of the construct. The cultivation of vital cells was possible with this method for more than 6 weeks. 

Machour et al. [71] and Szklanny et al. [79] took their approach from the current gold standard of free flap transplantation and developed a vascularized flap using 3D bioprinting. The construct combines a 3D bioprinted, self-assembled microvascular network with a mesoscale vascular scaffold, creating an implantable vascularized flap. The construct is composed of two parts that are fabricated separately. The microvascular network consists of recombinant human collagen methacrylate, human adipogenic microvascular endothelial cells (HAMECs), and dental pulp stem cells. The larger vessel is fabricated by 3D-printing a sacrificial mold filled with a 1:1 mixture of poly-L-lactic acid (PLLA) and polylactic-co-glycolic acid (PLGA). This is followed by freeze-drying of the scaffold, coating with fibronectin, and colonization with HAMEC. The vessel is then combined with the microvascular scaffold and initially cultivated in vitro. They showed that the endothelial cells of the two scaffolds anastomose in this process, and a vascular network is built. Subsequently, the scaffold was anastomosed with the femoral artery of a rat. In this way, complete and immediate perfusion of the construct was achieved. It was also shown that the host vessels of the rat connected to the construct, and the entire construct was perfused via anastomosis. Szklanny and colleagues also investigated whether engineered tissue can be supplied and cultured via the vascular network. For this purpose, they printed iPS-derived cardiomyocytes into the gel in addition to the cells for the formation of the network. The cardiomyocytes were cultured via perfusion in vitro and formed functional tissue. This was shown using troponin staining, contraction of the tissue, and calcium current visualized via the cells transfected with GCaMP. 

The studies described did not focus on specific tissue demands but generally investigated the formation of vascular networks. In the future, it will be relevant to combine the progress and findings for improved vascularization with tissue-specific TE. As discussed above, the choice of biomaterial is relevant for this, and the natural biomaterials used in many studies have limitations, particularly in terms of mechanical properties. For these reasons, the use of silk fibroin seems to be the obvious choice, as this material combines the advantageous properties of synthetic and natural materials [9]. Li et al. [80] developed a bioink consisting of alginate and fibroin to print a construct with perfusable hierarchical microchannels and improve the mechanical properties of alginate bioinks. They used a coaxial extrusion system with calcium ions and Pluronic F127 flowing through the core nozzle as crosslinkers. The printed scaffold had transversely and longitudinally interconnected microchannels, making it perfusable. The analysis showed that the combined constructs have a significantly higher compressive modulus than that of the alginate construct, at 16.0 kPa and 11.5 kPa, respectively. Additionally, the complex shear modulus is about 172.0 kPa for the alginate/fibroin construct, in contrast to 17.7 kPa for the crosslinked alginate alone. The construct was also tested for cytocompatibility and cell viability by bioprinting C3A cells (liver cancer cells). After cultivation for 14 days, the cell viability was 99.5% for the alginate/fibroin construct, compared to 88.4% for alginate.

The studies discussed demonstrate different approaches to engineered tissue vascularization. The most promising appear to be the approaches that combine different techniques, such as the vascularized flap of Szklanny and Machour [71,79]. However, current bioprinters lack a high-definition resolution that can be helpful in printing small vessels and other fine features within tissues, so indirect printing techniques or sacrificial structures are used [81,82]. Further technical improvements to printing systems, such as the FISH technique, may be able to overcome these limitations in the future. Despite the improvements and partly already larger constructs, vascularization has not yet succeeded in achieving the necessary sizes of constructs for application in humans.

**Table 3:** Summary of the reviewed literature related to vascularization.

Paper	Cell Type	Bioink	Experiment Type	Key Findings	Limitations
Sousa et al. (2021) [76]	HUVEC	Alginate (sacrificial) and photocrosslinkable glycidyl methacrylated xanthan gum (XG-GMA)	In vitro	Layer-by-layer-coated 3D-printed perfusable microchannels embedded in XG-GMA hydrogels	No in vivo investigation, upscaling needed
Shao et al. (2020) [77]	HUVEC, MC3T3-E1 (mouse osteoblast cell line)	GelMa, gelatin (sacrificial)	In vitro	Synchronous 3D bioprinting of cell-laden constructs with nutrient networks, construct size up to 3 × 3 × 3 cm^3^	Cultivation with osteoblast with no investigation for differentiation, no in vivo application
Machour et al. (2022) [71]	Human adipose microvascular endothelial cells + dental pulp stem cells	Recombinant human collagen methacrylate (rhCollMA) hydrogel, PLLA + PLGA	In vivo rat model	Hierarchical vessel network composed of microscale and mesoscale vasculatures, anastomosis with rat femoral artery	Proof of principle of the anastomosis, studies on successful in vivo tissue engineering pending, small size of the construct
Szklanny et al. (2021) [79]	Human adipose microvascular endothelial cells + dental pulp stem cells iPS-derived cardiomyocytes	Recombinant human collagen methacrylate (rhCollMA) hydrogel, PLLA + PLGA	In vitro	Supply of nutrients to differentiated and functional cardiomyocytes via the vascular network	
			In vivo rat model	Anastomosis with rat femoral artery	
Kolesky et al. (2016) [78]	HUVEC, hMSC *	Pluronic F-127 and thrombin (sacrificial); gelatin and fibrinogen	In vitro	Creation of thick human tissues (>1 cm) replete with an engineered extracellular matrix, embedded vasculature, and multiple cell types	No in vivo investigation, upscaling needed
Kreimendahl et al. (2021) [75]	HUVECs + HDFs	Fibrin + hyaluronic acid	In vitro	Use of FRESH printing technique: enables printing of low-viscose natural polymers with high shape stability, formation of a vascular network	No in vivo investigation, upscaling needed
Li et al. (2020) [80]	C3A	Alginate + silk fibroin	In vitro	Development of mechanically improved bioink. Scaffold with hierarchical microchannel network	No in vivo investigation, in vitro testing with cell line
Erdem et al. (2020) [74]	3T3 fibroblasts or rat cardiomyocytes	GelMa + CPO	In vitro	Development of a printable, O_2_ delivering biomaterial, cell viability under hypoxia was similar to normoxic conditions when CPO was added	No in vivo investigation, small size of the construct

* cell origin not stated

## 5. Conclusions

In conclusion, soft tissue engineering is a critical area of research for plastic and reconstructive surgery that offers an alternative to autologous transplantation and can reduce donor site morbidity and comorbidities. Scaffolds are crucial components of tissue engineering, providing a framework for cell attachment and growth and promoting endogenous tissue regeneration.

The benefits of 3D bioprinting in tissue engineering include the ability to create complex tissue constructs with high precision and reproducibility and the incorporation of different cell types and growth factors into the tissue construct. It allows for extensive control over the shape, porosity, size, and mechanical properties of the scaffolds. These advantages have led to great progress, especially in the field of skeletal muscle TE with its complex anatomy, where the interplay between tissue-specific cells, functionality, vascularization, and innervation is of great importance. These advantages can also be exploited for the TE of adipose tissue, particularly with regard to improving vascularization, although the significance of the new achievements through bioprinting seems less relevant in this respect.

Despite the many benefits of bioprinting and the progress it has made, significant challenges remain. One challenge is the choice of biomaterial, which should mimic the native ECM as much as possible in terms of biochemical and mechanical properties. The already-existing requirements are extended by the factor of printability. dECM is frequently used due to its many positive properties, as shown in the described studies. However, dECM hydrogels often lack the necessary strength. Silk fibroin, as a natural biomaterial with good mechanical properties, appears to be a promising material in this regard, although, to the best of our knowledge, it has not yet been applied for skeletal muscle or adipose tissue TE in combination with 3D bioprinting.

The use of 3D bioprinting also opens up new possibilities with regard to the vascularization of the constructs. In this context, the possibility of simultaneously printing several cell types with a precise spatial arrangement plays an important role and can lead to significantly improved results.

These advantages make 3D bioprinting a highly promising technology for the future of tissue engineering and regenerative medicine. Nevertheless, tissue constructs produced using 3D bioprinting remain in the order of a few centimeters, so there is still no breakthrough in sight in terms of clinical translation.

## Figures and Tables

**Table 1:** Summary of the reviewed literature related to skeletal muscle tissue engineering.

Paper	Cell Type	Bioink	Experiment Type	Key Findings	Limitations
Russell et al. (2020) [51]	C2C12	GelMa	In vitro	Successful differentiation, mechanical properties similar to skeletal muscle	Use of mouse cell line
	-		In vivo murine VML model	Proof of principle for the handheld printing device	No muscle regeneration in vivo
Kiratitanaporn (2022) [47]	C2C12	Poly(glycerol sebacate) acrylate + skmdECM coating	In vitro	Superiority of the scaffold coated with dECM	Secondary seeding of the scaffolds with limited infiltration
	-		In vivo rat VML model	Coating with dECM increased cellular infiltration, decreased fibrosis	Good cellular infiltration in the dECM-coated scaffold with limited muscle regeneration
Gokyer et al. (2021) [45]	C2C12	Thermoplastic polyurethane	In vitro	Development of a biocompatible and biodegradable, elastomeric, segmented TPU	Small size of the construct, vascularization not investigated
	hADSC		In vivo rat VML model	Comparison of cell-laden construct vs. acellular: more regeneration of muscle tissue in cellular construct in contrast to the acellular scaffold or the control group	
Choi et al. (2016) [48]	C2C12	skmdECM (porcine) + PCL	In vitro	Successful differentiation and parallel alignment	Use of mouse cell line, no in vivo evaluation
Choi et al. (2019) [55]	hSKM + HUVECs	skmdECM (porcine) + vdECM (Aorta descendens, porcine)	In vivo rat VML model	Coaxial nozzle enables the fabrication of a compartmentalized structure; improved de novo muscle fiber formation, vascularization, and innervation, 85% of functional recovery in VML injuries (compared to non-printed constructs)	Small size of construct, upscaling necessary for clinical application
Fornetti et al. (2023) [56]	Mabs or hSKM	PolyEthylene Glycol (PEG) fibrinogen	In vitro	Newly developed printing system for the use of PEG Fibrinogen	Printing system not explained. Presented results with mainly fibrotic tissue in vivo
			In vivo murine VML model		
Hwangbo et al. (2023) [57]	C2C12 or hADSC	GelMa	In vitro	Symbiotic co-cultivation with cyanobacteria (converting CO_2_ to O_2_) to reduce hypoxia, improvement of alignment of the cells through on-time electric stimulation while printing; combination of both led to increased myogenic differentiation in vitro + muscle regeneration in in vivo VML model using hADSC	Bacterial conversion through photosynthesis, light penetration through skin needed; implantation of bacteria in human muscle defects with high regulatory requirements
			In vivo murine VML model		
Fan et al. (2022) [28]	C2C12	Fibrinogen + gelatin	In vitro	Improved differentiation of thinner muscle bundles (0.6 mm vs. 2 + 5 mm)	Use of mouse cell line, innovation missing

GelMa—gelatin methacryloyl; PCL—polycaprolactone; Mabs—mouse mesangioblasts.

## Data Availability

Not applicable.
